# DNA Polymerase e - More Than a Polymerase

**DOI:** 10.1100/tsw.2003.08

**Published:** 2003-03-24

**Authors:** Helmut Pospiech, Juhani E. Syväoja

**Affiliations:** Biocenter Oulu and Department of Biochemistry, University of Oulu, Finland

**Keywords:** DNA polymerase epsilon, DNA replication, DNA repair, checkpoint, DNA damage response, chromatin

## Abstract

This paper presents a comprehensive review of the structure and function of DNA polymerase e. Together with DNA polymerases a and d, this enzyme replicates the nuclear DNA in the eukaryotic cell. During this process, DNA polymerase a lays down RNA-DNA primers that are utilized by DNA polymerases d and e for the bulk DNA synthesis. Attempts have been made to assign these two enzymes specifically to the synthesis of the leading and the lagging strand. Alternatively, the two DNA polymerases may be needed to replicate distinct regions depending on chromatin structure. Surprisingly, the essential function of DNA polymerase e does not depend on its catalytic activity, but resides in the nonenzymatic carboxy-terminal domain. This domain not only mediates the interaction of the catalytic subunit with the three smaller regulatory subunits, but also links the replication machinery to the S phase checkpoint. In addition to its role in DNA replication, DNA polymerase e fulfils roles in the DNA synthesis step of nucleotide excision and base excision repair, and has been implicated in recombinational processes in the cell.

## DISCOVERY AND STRUCTURE OF POL ε

DNA polymerase (Pol) ε was first isolated as a nuclear Pol from yeast as early as 1970[1]. Soon after, the corresponding Pol was purified from rabbit bone marrow as an enzyme associated with 3′→5′ exonuclease activity[2]. It has been isolated and characterised from several mammalian sources as an enzyme with varying molecular weights, 170 kDa from human placenta[3], ~260 kDa from HeLa cells[4,5,6], and 140 kDa from calf thymus[7,8]. At least for calf thymus Pol ε, it was shown that the 140-kDa form represents a proteolytic fragment, and that a similar fragment can be formed specifically during apoptosis by caspase-3 and calpain[9,10]. Pol ε was initially considered to be a form of Pol δ that was processive in the absence of PCNA and RFC at low ionic strength[11]. Later Pol ε was shown to be an independent Pol and the catalytic subunit was isolated and cloned from several species[12,13,14,15,16,17,18,19,20].

Pol ε is a member of the B family of Pols[21,22] that includes the two other replicative Pols, α and δ. The structure of Pol ε is typical of this family, but notably, the catalytic subunit possesses a large C-terminal domain of unclear function that accounts for half of the molecular weight of ~260 kDa[23]. This domain is located between the Pol domain and the putative zinc-finger.

Detailed phylogenetic analyses indicate that Pol ε is the orthologue to the archaeal Pol BI rather than to Pols α, δ, or ζ[24,25]. Therefore, a gene duplication event of the ancestral family B Pol gene must have occurred early in evolution prior to the separation of the eukaryotic and archaeal lineages[24]. Pol B1 is found ubiquitously in Archaea and, like the eukaryotic Pols of family B, is inhibited by aphidicolin[24,26,27]. The phylogenetic affiliation of Pol ε with the archaeal Pols is also reflected by several similarities in the primary sequence of these enzymes[23]. Whereas the five typical motifs of the exonuclease domain are well conserved in Pol ε[28,29,30], several changes in seven conserved motifs that characterise the Pol domain are distinct in Pol ε compared to other family B Pols[23,31,32,33]. The changes mainly concern residues that have been implicated in template-primer binding and stabilisation of the newly incoming nucleotide, arguing for a catalytic centre architecture that is distinct from other Pols of family B.

Apart from the large catalytic subunit, Pol ε contains three more subunits that differ in size but are conserved in their primary structure from yeast to human (Table 1, Fig. 1, [34]). The second largest, subunit B, has been cloned from several species[35,36,37]. Like the catalytic subunit[13,18,19], the B subunit is also essential for viability in yeast[35]. The *DPB3* and *DPB4* genes encode the two smallest, nonessential subunits of budding yeast Pol ε[38,39]. The fourth subunit p17 of the human Pol ε, homologous to *DPB4*, has been cloned, and the probable *DPB3* homologue p12 has been identified by homology searches[40]. Initially, a subunit stoichiometry of 1:1:4:4 has been proposed for purified as well as reconstituted recombinant yeast Pol ε[41,42]. A recent, detailed analysis of the properties of native yeast Pol ε indicates that the enzyme is rather a heterotetramer with a stoichiometry of 1:1:1:1[43].

## STRUCTURE AND FUNCTION OF THE B SUBUNIT

Despite their variable size of 59–86 kDa, the Pol ε B subunits are homologous in primary structure[35,36,37]. More detailed analysis of the human B subunit revealed further similarities to B subunits of the eukaryotic Pols α and δ, as well as to the small subunit of the archaeal Pol DI (Pol II)[44]. Twelve conserved motifs cover most of the primary structure of the B subunits and define a protein superfamily (Fig. 2). It is particularly intriguing that the catalytic subunit associated with eukaryotic B subunits belong to the Pol family B, whereas the catalytic subunit associated with archaeal B subunits belong to family D. Enzymes of this Pol family have so far only been identified in the kingdom Euryarchaeota, and they do not share sequence homology to other Pol families[27,45,46]. The conservation of the B subunits of different Pols argues not only for a similar fold, but also for a conserved function shared by these subunits. All Pols with conserved B subunits are implicated in DNA replication[34,48] and they are all essential for cell viability[49]. Genetic and biochemical studies indicate that the putative zinc finger regions in the catalytic subunit of Pols δ and ε are important for the interaction with the B subunits[47,50,51].

**TABLE 1**
**Subunit Designation of Pol ε from Different Species**

| Subunit Designation | Human | *S. cerevisiae* | *S. pombe* | Comments |
|---|---|---|---|---|
| A | **p261 (*POLE*)**<br>(262)[1] | ***POL2***<br>(256) | **cdc20$^{+}$**<br>(253) | Catalytic subunit; DNA polymerase and proofreading exonuclease |
| B | **p59 (*POLE2*)**<br>(60) | ***DPB2***<br>(79) | **dpb2$^{+}$ (SPBP8B7.14c)**[2]<br>(67) | Interacts with subunits A; forms a dimer; potential OB-fold and phosphoesterase-like domains |
| C | **p12**[2]<br>(12) | ***DPB3***<br>(23) | **SPAC17G8.03c?**[2,3]<br>(22) | Interacts with subunits A and D; histone-fold |
| D | **p17 (*POLE3*; *CHRAC17*)**<br>(17) | ***DPB4***<br>(22) | **dpb4$^{+}$ (SPBC3D6.09)**[2]<br>(24) | Interacts with subunits A and C; subunit of chromatin remodeling complex CHRAC; histone-fold |

1The predicted molecular weight in kDa is indicated in brackets.

2Assignment based on sequence homology.

3The DPB3 homologue of *S. pombe* cannot be assigned unambitiously by sequence homology.

No catalytic function has been assigned to the Pol ε B subunit or other members of the family. Nevertheless, a calcineurin-like phosphoesterase domain spanning the C-terminal half of the protein has been identified[52,53,54,55], as well as an OB(oligonucleotide/oligosaccharide binding)-fold domain in archaeal B subunits[56,57] (Fig. 2). The OB-fold and the calcineurin-like domain are separated by a short proline-rich region[44]. The OB-fold suggests that nucleic acid binding may be a common feature of Pol B subunits.

The presence of a calcineurin-like domain suggests that an enzymatic activity was originally intrinsic to B subunits that has been lost in Pol ε and other eukaryotic B subunits[52,53,54,55]. The B subunits may therefore represent an ancient proofreading exonuclease that is still present in the Pol family D[58].

The Pol ε B subunit has been implicated in protein-protein interactions. It mediates homodimerisation of the enzyme and interacts with the C-terminal part of the catalytic subunit as well as with the two small subunits[51].

Recently, the mouse Pol ε B subunit was found to interact with SAP18, which is known to associate with the transcriptional corepressor Sin3[59] (Fig. 1). Sin3 is a component of a protein complex possessing histone deacetylase activity that interacts with several transcriptional corepressors and functions in transcriptional silencing[60]. Recruitment of the B subunit/Sin3 complex to DNA resulted in transcriptional repression in a model system. The effect depended at least in part on the histone deacetylase activity[59]. It has been proposed that Sin3 is involved in the establishment of growth arrest by repressing transcription from E2F-dependent promoters and possibly alters chromatin structure at DNA replication origins[61]. These observations can be linked to the unpacking and repacking of chromatin during DNA replication or its initiation. On the other hand, the interaction with the histone deacetylase complex could also be important for the regulation of Pol ε function itself due to potential covalent modification of the two smallest subunits that possess a histone fold (see below).

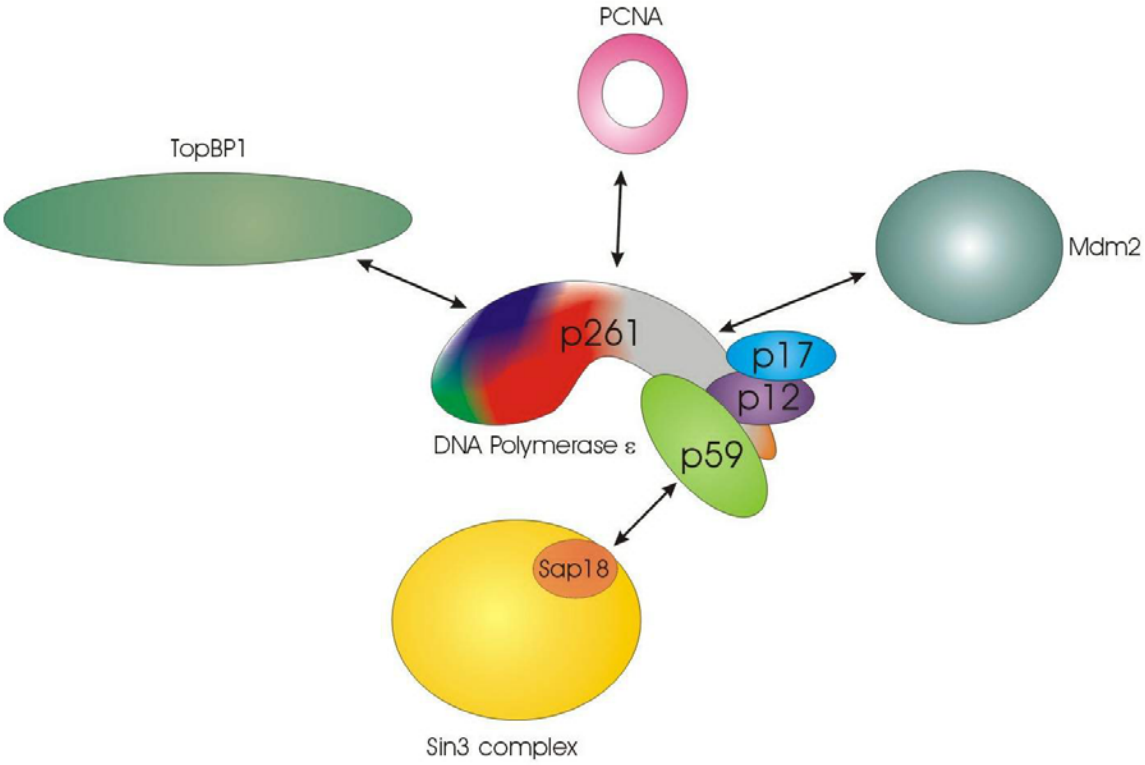

**FIGURE 1**. Subunit structure of human Pol ε, and the reported interactions with other proteins. The aminoterminal, exonuclease, and polymerase domains, and the carboxyterminal zinc fingers of the catalytic subunit are indicated schematically in green, blue, red, and orange, respectively.

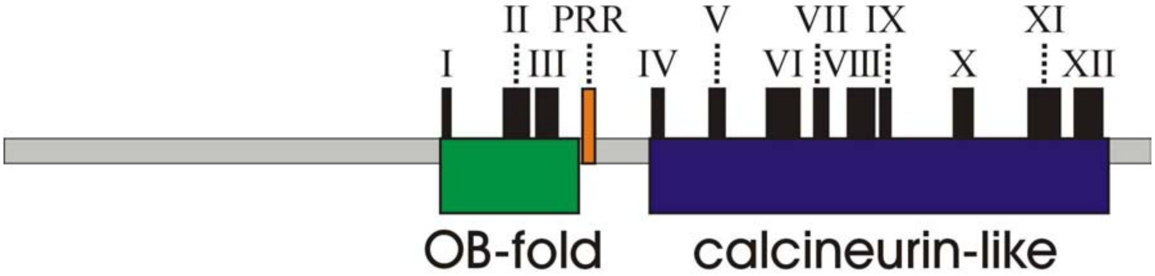

**FIGURE 2.** General structure of the Pol-associated B subunits. The location of the conserved motifs is indicated on top and marked with Roman numerals. PRR designates the proline-rich region between motifs III and IV. Below, the domain architecture of the B subunits is depicted.

In the budding yeast *Saccharomyces cerevisiae*, Pol ε has a role in the silencing of ribosomal DNA at the mating type locus, as is also the case for Pol α and several other replication factors[62,63]. The silenced DNA is characterised by a low content of histone acetyl groups. The underlying mechanisms that link replication factors to transcriptional silencing are not yet understood, and although silencing requires transition through the S phase of the cell cycle, the process is not dependent on DNA replication itself[64,65]. Clearly, very little is known of the details that link Pol ε to transcriptional regulation.

## THE TWO SMALLEST SUBUNITS OF POL ε - THE CHROMATIN CONNECTION

The two smallest subunits of Pol ε possess a histone-fold of the H2A/H2B type and are closely related to the histone-fold subunits of the CCAAT binding factor[39,40]. They form a stable dimer that interacts with the other two larger subunits[40,51]. The behaviour of the smaller subunits on SDS polyacrylamide gels suggests a posttranslational modification. In particular, the yeast Dpb3p with a calculated molecular weight of 23 kDa, migrates as two discrete bands of 31 and 34 kDa. Apart from phosphorylation, the subunits could be subject to acetylation and methylation that are typical for histone-like proteins.

Interestingly, p17 has been independently identified as an integral subunit of the chromatin-remodelling factor CHRAC[66]. CHRAC is an ISWI containing complex identified in different species that regulates chromatin accessibility and nucleosome spacing[66,67]. In CHRAC, Pol ε p17 forms a dimer with a protein closely related to the Pol ε p12 subunit that can interact with histones and DNA[68]. Such a homologue of *DPB3* was also identified in yeast[69].

## INTERACTION OF POL ε AND PCNA

The functional interaction between Pol ε and PCNA has been the subject of a detailed study. Like Pol δ, Pol ε forms a highly processive holoenzyme complex with replication factors RFC and PCNA under physiological conditions[6,70,71]. The interaction of Pol ε with PCNA apparently serves a dual role in stimulating the activity of the enzyme. PCNA facilitates binding of the primer end and prevents unproductive binding of single-stranded DNA[71,72,73]. On the other hand, PCNA stimulates the activity of the enzyme itself. The interaction sites of PCNA for Pol ε have been mapped in detail by mutational analysis of PCNA. While for Pol δ, the interaction with the interdomain loop of PCNA appears to be most important, Pol ε interacts with the front side of PCNA including its very C-terminus[74,75,76]. A second interaction with the back is important for PCNA stimulation of the primer binding by Pol ε[76]. The PCNA interaction sites of Pol ε are not so well characterised. A near-consensus PCNA-interacting protein (PIP) site[77] has been identified in the primary structure of the catalytic subunit adjacent to the Pol domain[42,78] (Fig. 1). A peptide corresponding to this motif in human Pol ε is able to bind PCNA, albeit with low efficiency (A. Rytkönen, J. Tuusa, and J.E. Syväoja, unpublished observation). Mutation of the motif in budding yeast did not affect cell viability, but rendered the cells sensitive to methyl methanesulphonate. This suggests that the region is actually involved in the interaction with PCNA. Supportive evidence again comes from the related archaeal Pols of family B. Many of these possess a related PIP motif at the same position as in Pol ε, adjacent to the conserved motif C-3[23,79] (Fig. 3). This site is located in the very C-terminus of the archaeal Pols. Archaeal Pols have been shown to bind PCNA, resulting in increased activity due to higher processivity[79,80,81]. Despite the functional interaction with PCNA, one should bear in mind that Pol ε retains considerable activity and processivity even in the absence of PCNA under various reaction conditions[5,82]. This is a fundamental difference from Pol δ and it is possible that Pol ε performs limited DNA synthesis in the absence of PCNA in the cellular context[72].

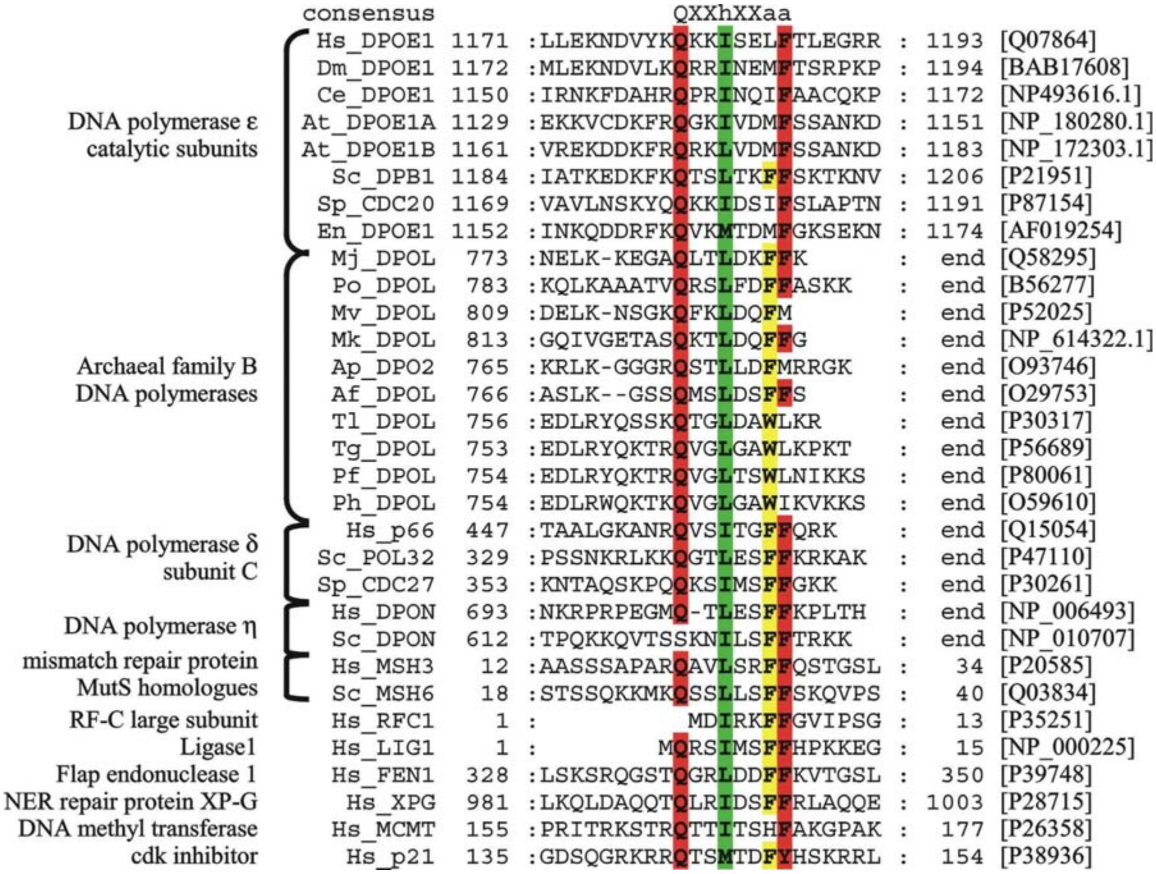

**FIGURE 3**. A conserved PCNA interaction site among Pol ε and archaeal Pols of family B. The conserved PCNA-interacting sites of Pol ε, archaeal Pols of family B, Pol δ subunit C, Pol η, as well as representative replication and cell cycle regulating proteins are aligned. Species abbreviations are Hs – *Homo sapiens*; Dm – *Drosophila melanogaster*; Ce – *Caenorhabditis elegans*; At – *Arabidopsis thaliana*; Sc – *Saccharomyces cerevisiae*; Sp – *Schizosaccharomyces pombe*; En – *Emericella nidulans*; Mj – *Methanococcus jannaschii*; Po – *Pyrodictium occultum*; Mv – *Methanococcus voltae*; Mk – *Methanopyrus kandleri*; Ap – *Aeropyrum pernix*; Af – *Archaeoglobus fulgidus*; Tl – *Thermococcus litoralis*; Tg – *T. gorgonarius*; Pf – *Pyrococcus furiosus*; Ph – *P. horikoshii*. Genbank accession numbers are indicated next to each sequence.

## POL ε IS INVOLVED IN REPLICATION OF CELLULAR DNA

The introduction of Simian Virus 40 (SV40) DNA replication as a model for mammalian DNA replication by Thomas Kelly and coworkers[83] allowed the rapid identification and characterisation of cellular factors involved in replication. The SV40 replisome can be considered the prototypic core replication apparatus in the eukaryotic cell, with the exception that the viral large T antigen serves both as initiator and as replicative DNA helicase[84]. Pols α and δ are involved both in leading and lagging strand DNA synthesis in the SV40 DNA replication system reconstituted with purified proteins[85,86,87]. The switch from Pol α to Pol δ occurs after initiation of the leading strand as well as after priming of each Okazaki fragment on the lagging strand with the primase of the Pol α-primase complex[88,89,90]. Pol ε is not required in this system[6,89,90,91,92].

The validity of the SV40 DNA replication system as a model of cellular DNA replication is challenged by research done with cellular systems. The catalytic subunit of Pol ε is essential for viability in budding and fission yeast, and temperature-sensitive mutants fail to replicate chromosomal DNA at the restrictive temperature[13,18,19,93,94]. It is rather surprising though, that in fact the carboxy-terminal domain is sufficient for cell viability, whereas the Pol and exonuclease domains are dispensable[95,96,97]. The putative zinc fingers appear to be particularly important, since several mutants in this domain or the adjacent C-terminus have growth defects[50,94]. The results could be explained if the Pol ε carboxy-terminal domain is required for the assembly of the cellular replication apparatus while the catalytic domains are not essential for replication, or possibly the catalytic function of Pol ε could be replaced by other Pols, most likely by Pol δ. Nevertheless, another Pol activity cannot fully substitute Pol ε, since the deletion of the N-terminal portion of the enzyme causes temperature sensitive phenotype in a certain strain background, abnormal S phase progression and delayed growth, shortening of telomeres, and accelerated senescence[95,96,97,98]. It is therefore apparent that in normal cells, the catalytic domains are involved in replicative DNA synthesis. This has been shown to be the case in human cells where Pol ε can be cross-linked to nascent cellular DNA within replicating chromosomes[91] and replicative DNA synthesis is inhibited when neutralizing antibodies against Pol ε are introduced into isolated nuclei or nuclei of growing cells[92]. In line with this, removal of Pol ε from a *Xenopus* egg extract resulted in aberrant chromosomal DNA replication[99].

These results not only confirm the important role of Pol ε in nuclear DNA replication, but also indicate that the enzyme actually synthesises a significant fraction of new DNA in proliferating cells. If the Pol domain is not deleted, but inactivated by mutation of the catalytic centre, yeast cells are rendered deficient in replication and therefore inviable[96]. Similarly, several yeast temperature sensitive mutants of Pol ε map to the catalytic domain, and arrest replication at the restrictive temperature[93]. This raises the possibility that the N-terminal part of Pol ε blocks or disrupts the replication fork when present but inactive, but may be substituted for when it is completely absent.

## WHAT IS THE ROLE OF POL ε IN REPLICATION?

Several approaches have been utilised to address the division of labour between Pols δ and ε in cellular DNA replication (Table 2). *S. cerevisiae* Pol ε associates with origin DNA before the initiation of DNA replication. Upon initiation, it dissociates from the origin and associates with nonorigin DNA, with kinetics similar to those of Cdc45 and MCM proteins, probably following the replication fork[100].

A role in elongation and maturation of the lagging strand was proposed for Pol ε based on biochemical properties[6]. Analysis of the phenotype of exonuclease deficient mutants of Pols δ and ε showed that both Pols function consecutively with mismatch repair, i.e., in DNA replication. This finding led A. Sugino to propose that Pol ε could function in leading strand synthesis, whereas Pol δ would perform the bulk of lagging strand synthesis[13,101]. This view was supported by more detailed analysis of the exo$^-$ mutants placing marker genes in opposite directions in the vicinity of a replication origin in yeast[102]. In this elegant approach, mutational analysis of the marker gene in different Pol exo$^-$ backgrounds indicates that only one, but not both strands, is encountered by the defective Pol[103,104]. This again placed the two enzymes on different DNA strands. However, Datta and coworkers[105] demonstrated that the Pol δ exo$^-$ mutator phenotype depends on the S phase damage checkpoint pathway, and is not a direct consequence of inaccurate DNA synthesis. There is a clear strand bias for replication errors on leading and lagging strand established by yeast origins[106]. It remains to be studied whether this bias is due to different fidelities of the two Pols at the replication fork. Burgers[49] proposed, as a modification of his earlier model, that Pol δ would elongate DNA both on the leading and lagging strands, whereas Pol ε would only be required for lagging strand maturation. This model assumes that Pol ε synthesises less DNA than Pol δ, based predominantly on the less severe phenotype of

## TABLE 2
## Proposed Functions of Pol ε and Their Dependence on the Catalytic Activity of the Enzyme

| Function | DNA Polymerase Activity Required | Evidence | References |
|---|---|---|---|
| **Nuclear DNA replication** | Involved but not essential | Genetic analysis in *S. cerevisiae* and *S. pombe* | [13,18,19, 35,93,94 95,96] |
| | | Cross-linking af nascent DNA to Pols | [91] |
| | | Microinjection of antibodies into human fibroblasts | [92] |
| | | Replication in isolated human cell nuclei | [92] |
| | | Immunodepletion of *Xenopus* egg extracts | [99] |
| Initiation of replication | Not determined | Biochemical analysis in *S. cerevisiae* and *Xenopus* egg extracts | [100,123, 124,130] |
| Replication of leading or lagging strand | Yes | Analysis of exonuclease-deficient mutants in *S. cerevisiae* | [103,104] |
| Replication of heterochromatin | (Yes)[1] | Immunofluorescence microscopy in human fibroblasts | [122] |
| | | Histone-fold subunit shared with chromatin remodelling factor | [39,40,66, 68] |
| **DNA repair** | | | |
| Nucleotide excision repair | Yes | Reconstitution of the human repair pathway | [135,137, 139] |
| | | Biochemical analysis in *S. cerevisiae* | [142,143, 144] |
| Base excision repair | Yes | Reconstitution of the human repair pathway | [153,158] |
| | | Biochemical analysis in *S. cerevisiae* | [140] |
| **Recombination** | (Yes)[1] | Part of mammalian recombination complex I mediating stand exchange | [120] |
| | | Purifies with a *Xenopus* non-homologous end joining activity | [163] |
| | | Genetic analysis in *S. cerevisiae*; | [110,111] |
| **DNA damage response** | No | Genetic analysis in *S. cerevisiae* | [50,125,126] |
| **Transcriptional silencing** | No | Genetic analysis in *S. cerevisiae* | [62,63] |
| | | Interaction of mouse B subunit with Sin3 co-repressor complex | [59] |

1A requirement for the polymerase activity of Pol ε for replication of heterochromatin or during recombination processes seems likely, but has not been directly proven.

Pol ε compared to Pol δ exo$^-$ mutants. Since this assumption is no longer supported, and since Pol δ has been shown to be actively involved in lagging strand maturation[107,108], the Burgers model lacks conclusive evidence. An explanation of the role of Pol ε should take into account differences between cellular DNA replication and the SV40 system. The larger eukaryotic genome necessitates more complex cell cycle and DNA damage checkpoint control. Pol δ appears to have an important role in replicative damage bypass by translesion synthesis[105,109].

Pol ε is not only important during the initiation of DNA replication[100], but also in the recombinational processes during S-phase[110,111]. Such processes are induced by DNA breaks that may be normal and common in replicating chromosomes[110,112]. Although break-induced recombination may be explained in several ways, models that involve the formation of a replication fork are most likely[113]. These models closely resemble the mechanisms for recombination-induced late replication of the T4 phage and recombination-dependent replication in *E. coli*[114,115,116]. It appears that break-induced recombination is particularly important for the maintenance of telomeres in the absence of telomerase, and for the restart of DNA replication at broken replication forks[116,117,118]. Pol ε seems to play a role in establishing a DNA synthesome during gene conversion[111]. Similarly it may be required for recombination-dependent DNA replication processes and it is likely that Pols δ and ε cannot substitute for each other in these specific functions. Consistent with its possible role in recombination-induced replication, Pol ε has been found to be a component of a human recombination complex that is able to repair deletions and double-strand breaks of DNA[119,120]. It also seems to play a specific role in completion of meiotic recombination in mouse testis[121].

The recent finding that Pol ε forms nuclear foci that colocalise with sites of DNA replication in late, but not early S phase, provides another potential solution for the division of labour between Pols δ and ε[122]. These results indicate that the chromatin structure could determine which Pol replicates certain DNA; Pol δ would be responsible for the replication of more open chromatin in early S phase whereas Pol ε would replicate more compact heterochromatin in late S phase. Support for this view seems to come from analysis of the primary structure of Pol ε that provides several links to chromatin structure and remodelling. These results, however, do not explain why Pol ε associates similarly with early and late firing origins in budding yeast, and is associated with the very open embryonic chromatin in *Xenopus* egg extracts in a replication-dependent manner[123,124]. Different models and novel approaches will be required to resolve conclusively the specific roles of Pols δ and ε during cellular replication.

## THE ROLE OF POL ε IN THE DNA DAMAGE RESPONSE

Besides DNA replication, the putative zinc finger domain or the adjacent C-terminus is involved in the response to DNA damage, since some of the budding yeast mutants with mutations in this region are defective in their response to DNA damaging agents during S-phase[50,125,126]. Further evidence of the checkpoint function of budding yeast Pol ε comes from identification of the *DPB11* gene as a multicopy suppressor of the temperature-sensitive *Pol2* (encodes the catalytic subunit of Pol ε) and *DPB2* (encodes the B subunit of Pol ε) mutants of Pol ε[127]. The gene product is homologous to the fission yeast Cut5, both having four copies of the BRCA1 C-terminal (BRCT) domain. Dpb11 and Cut5 are required for the onset of S-phase, DNA replication and cell cycle checkpoints[127,128,129]. Dpb11 also interacts physically with Pol ε, but this interaction appears to be transient and may be restricted to initiation of replication[130]. The topoisomerase IIβ binding protein 1 (TopBP1) contains eight BRCT domains and is a structural and functional human homologue of Dpb11. It is required for DNA replication; it binds to Pol ε (Fig. 1) and relocates to stalled replication forks in response to replication blocks[131,132]. It also localises to sites of DNA double strand breaks and is phosphorylated in response to the damage. This phosphorylation depends on the ataxia telangiectasia mutated protein (ATM) *in*

*vivo*[131,132]. Recent work indicates that TopBP1 may represent an interaction platform for proteins that function in cell cycle regulation.

In contrast to the situation in budding yeast, there is no evidence for a direct involvement of Pol ε in DNA checkpoint control in the fission yeast *Schizosaccharomyces pombe*[18]. Instead, the Pol σ homologue Cid1 is required for S-M checkpoint control when Pol δ or ε is inactivated, or DNA replication is blocked[133].

The ubiquitin ligase and oncoprotein MDM2 was found to interact with the C-terminal region of human Pol ε[134] (Fig. 1). The regulatory and functional implications have not yet been studied, but one could envision that MDM2 is sequestered by Pol ε in response to DNA damage, thereby stabilising p53. On the other hand, Pol ε could be targeted by MDM2 for proteosome-dependent degradation.

## POL ε IN DNA REPAIR

Pol ε was originally isolated from HeLa cells as a factor required for the repair of UV damage in human fibroblasts[4]. Nucleotide excision repair (NER) represents the major repair pathway for this type of DNA damage. In a reconstituted system utilising UV-irradiated DNA and a mixture of purified and recombinant proteins, both calf thymus Pol δ and the 140-kDa form of Pol ε could perform the gap-filling DNA repair synthesis[135]. This is consistent with studies using model DNA substrates containing short gaps[72,136,137,138]. The Pol requirement of nucleotide excision repair was re-examined utilising highly purified recombinant incision components and highly purified human Pols[139]. DNA repair synthesis of a defined cis-platin adduct occurred also with Pol δ and Pol ε, and depended on PCNA and RFC for both enzymes.

The powerful genetics of budding yeast has been used extensively to analyse the Pol requirement of UV damage repair. These studies, however, suffer in part from the fact that several DNA repair pathways operate in parallel in yeast to correct this type of damage. Wang and coworkers[140] found a role for Pol ε in the repair of UV damaged plasmids in *S. cerevisiae* nuclear extracts in an experimental set-up very similar to the one described earlier. The authors could demonstrate that their repair depended on base excision repair (BER) rather than NER[141]. Analysis of changes in the molecular weight of cellular DNA after UV irradiation in various mutant backgrounds was used to determine the Pol requirement of the repair[142,143,144]. In all studies, Pol δ and Pol ε were implicated in the filling of repair gaps that were formed in a Rad1 (NER)-dependent manner, but whereas the former study indicated redundancy of the two Pols, the latter two suggested that the presence of both enzymes is required for repair of the UV lesions.

In mammalian BER, either a short-patch or a long-patch repair pathway may be utilised[145,146,147,148]. In short patch BER, which appears to be the major BER pathway, a gap of usually only one nucleotide is created during repair and filled by Pol β[145,149]. The repair is then completed by sealing the remaining nick utilising the complex of DNA ligase III and XRCC1[149,150]. DNA ligase I may possibly substitute for the XRCC1/ligase III in some instances[147,151]. According to recent reports, Pol β also initiates regular long-patch BER, which involves synthesis of 2-8 nucleotides beginning at the damage site[146,152,153,154,155,156,157]. Poly(ADP-ribose) polymerase-1 (PARP-1) is required for a switch to initiate long-patch BER when the repair product cannot be ligated after incorporation of the first nucleotide by Pol β[155,156,157]. In this case, Pol β is replaced by Pols δ or ε, which then conducts strand displacement synthesis[153,158]. DNA synthesis is directed by the DNA sliding clamp PCNA and the clamp loader RFC, and the displaced DNA is removed as an oligonucleotide by the flap endonuclease Fen-1[159,160]. Repair is completed by DNA ligase I or alternatively by XRCC1/ligase III. PCNA probably has an important role in coordination of this pathway since it interacts with Pol δ/ε, Fen-1, and DNA ligase I[152,160,161,162].

Besides the role in homologous recombination processes that have already been discussed, Pol ε has also been identified in a partially purified nonhomologous end-joining (NHEJ) activity[163]. This activity probably represents a minor NHEJ pathway not dependent on DNA-dependent protein kinase that joins noncomplementary DNA ends without a requirement for homology, but with the generation of deletions. In addition to Pol ε, the 5′-specific FEN-1, DNA ligase III, and possibly PCNA are involved in this pathway[163,164].

While Pols δ and ε appear to be largely redundant during NER and long patch BER, these two Pols also have nonredundant functions in DNA repair. As described, Pol ε has been repeatedly implicated in recombinational processes, whereas Pol δ apparently fulfils specific functions during mismatch repair as well as postreplicational repair by DNA translesion synthesis[109,165,166].

## CONCLUSIONS

Pol ε is an enzyme of both complex structure and function. As presented in this review, Pol ε shares several structural and functional features with archaeal Pols of family B on the one hand, and with the related eukaryotic replicative Pol δ on the other. However, Pol ε differs from these Pols in that it possesses a large C-terminal domain that is not required for its enzymatic function. It appears that this domain mediates many of the functions of Pol ε, and it is this region, but not the Pol domain, that renders Pol ε indispensable for cell viability. Even 30 years after its first isolation, Pol ε still offers surprises. Understanding the function of Pol ε is a prerequisite for understanding the mechanisms of eukaryotic DNA replication and the maintenance of genome integrity in general.

## ACKNOWLEDGEMENTS

J.E.S. is supported by the Academy of Finland and the Cancer Society of Finland.
